# The Complete Mitochondrial Genome of *Corizus tetraspilus* (Hemiptera: Rhopalidae) and Phylogenetic Analysis of Pentatomomorpha

**DOI:** 10.1371/journal.pone.0129003

**Published:** 2015-06-04

**Authors:** Ming-Long Yuan, Qi-Lin Zhang, Zhong-Long Guo, Juan Wang, Yu-Ying Shen

**Affiliations:** State Key Laboratory of Grassland Agro-Ecosystems, College of Pastoral Agricultural Science and Technology, Lanzhou University, Lanzhou, Gansu, People's Republic of China; University of the Sunshine Coast, AUSTRALIA

## Abstract

Insect mitochondrial genome (mitogenome) are the most extensively used genetic information for molecular evolution, phylogenetics and population genetics. Pentatomomorpha (>14,000 species) is the second largest infraorder of Heteroptera and of great economic importance. To better understand the diversity and phylogeny within Pentatomomorpha, we sequenced and annotated the complete mitogenome of *Corizus tetraspilus* (Hemiptera: Rhopalidae), an important pest of alfalfa in China. We analyzed the main features of the *C*. *tetraspilus* mitogenome, and provided a comparative analysis with four other Coreoidea species. Our results reveal that gene content, gene arrangement, nucleotide composition, codon usage, rRNA structures and sequences of mitochondrial transcription termination factor are conserved in Coreoidea. Comparative analysis shows that different protein-coding genes have been subject to different evolutionary rates correlated with the G+C content. All the transfer RNA genes found in Coreoidea have the typical clover leaf secondary structure, except for *trnS1* (AGN) which lacks the dihydrouridine (DHU) arm and possesses a unusual anticodon stem (9 bp vs. the normal 5 bp). The control regions (CRs) among Coreoidea are highly variable in size, of which the CR of *C*. *tetraspilus* is the smallest (440 bp), making the *C*. *tetraspilus* mitogenome the smallest (14,989 bp) within all completely sequenced Coreoidea mitogenomes. No conserved motifs are found in the CRs of Coreoidea. In addition, the A+T content (60.68%) of the CR of *C*. *tetraspilus* is much lower than that of the entire mitogenome (74.88%), and is lowest among Coreoidea. Phylogenetic analyses based on mitogenomic data support the monophyly of each superfamily within Pentatomomorpha, and recognize a phylogenetic relationship of (Aradoidea + (Pentatomoidea + (Lygaeoidea + (Pyrrhocoroidea + Coreoidea)))).

## Introduction

The insect mitochondrial genome (mitogenome) is a circular double-strand molecule of 15–18 kb in size and usually codes for 37 genes: 13 protein-coding genes (PCGs), two ribosomal RNA genes (rRNAs), and 22 transfer RNA genes (tRNAs) [1, 2]. In addition, mitogenome usually contains a large non-coding region, known as control region (also called A+T-rich region in insects due to high A+T content). This region contains essential regulatory elements for transcription and replication [1, 3], and has been identified as the source of size variation in the whole mitogenome [4]. Compared to single mitochondrial gene, mitogenome contains even more genetic information and provides genome-level features (e.g. gene rearrangements and RNA secondary structures) [5–8]. Due to its maternal inheritance, relatively rapid evolutionary rate, and lack of genetic recombination, mitogenome sequences have been extensively used in the study of molecular evolution, phylogenetics, phylogeography and population genetics [2, 9–11].

Pentatomomorpha, which consists of 40 families representing more than 14,000 species, is the second largest among the seven infraorders of Heteroptera [12]. Most members of this group are phytophagous, and economically important in agriculture and forestry. Currently, the classification system of Pentatomomorpha includes five superfamilies (Aradoidea, Pentatomoidea, Coreoidea, Lygaeoidea and Pyrrhocoroidea), and the superfamilies except the Aradoidea are grouped as Trichophora [12, 13]. Based on morphological and molecular evidence, the hypothesis of (Aradoidea + (Pentatomoidea + the remainder of Trichophora)) has been accepted by most researchers [13–17]. However, the phylogenetic relationships among the superfamilies within the Trichophora are still controversial.

To date, complete or nearly complete mitogenomes have been determined for 25 species from Pentatomomorpha (GenBank, September 10, 2014), of which four are from Coreoidea. In this study, we sequenced and annotated the complete mitogenome sequence of *Corizus tetraspilus* (Hemiptera: Rhopalidae), an important pest of alfalfa in China. We analyzed the main features of *C*. *tetraspilus* mitogenome, including nucleotide composition, codon usage, rRNA structures and evolutionary pattern of PCGs, and provided a comparative analysis with four other Coreoidea species. To investigate the phylogenetic relationships among the superfamilies of Pentatomomorpha, we also performed phylogenetic analyses with Bayesian inference (BI) and maximum likelihood (ML) methods using the concatenated nucleotide sequences of 13 mitochondrial PCGs and 24 RNA genes.

## Materials and Methods

### Ethics Statement

No specific ethics permits were required for the described studies. The insect specimens were collected from alfalfa field by net sweeping, and no specific permissions were required for these locations/activities. The species in our study is an agricultural pest and are not included in the ‘‘List of Protected Animals in China”.

### Sample and DNA Extraction

Adult specimens of *C*. *tetraspilus* were collected from alfalfa field in Shishe Town, Xifeng District, Qingyang City, Gansu Province, China, in July 2013. Samples and voucher specimens have been deposited in the State Key Laboratory of Grassland Agro-Ecosystems, College of Pastoral Agricultural Science and Technology, Lanzhou University, Lanzhou, China. All specimens were initially preserved in 100% ethanol in the field, and transferred to -20°C until used for DNA extraction. The total genomic DNA was extracted from thorax muscle of a single specimen using the OMEGA Insect DNA Kit (OMEGA, USA) according to the manufacturer’s protocols.

### PCR Amplification and Sequencing

The whole mitogenomes of *C*. *tetraspilus* were amplified with ten overlapping fragments by using universal insect mitogenome primers [9] and species-specific primer pairs designed from sequenced fragments (S1 Table). PCRs were performed with TaKaRa LA Taq under the following conditions: 2 min initial denaturation at 92°C, followed by 35 cycles of 10 s at 92°C, 1 min at 48–55°C, and 1–3 min at 68°C, and a final elongation for 10 min at 68°C. All PCR products were electrophoresed on a 1.2% agarose gel, purified, and then directly sequenced or cloned into the pEASY-T1 vector (TransGen Biotech, Beijing, China). All fragments were sequenced in both directions on an ABI3730 automated sequencer.

### Annotation and Sequence Analysis

Sequence files were proof read and assembled into contigs with BioEdit 7.0.9.0 [18]. PCGs were identified by ORF Finder implemented at the NCBI website with the invertebrate mitochondrial genetic codes. To ensure the accurate boundaries of different genes, PCGs and rRNAs were aligned with the sequenced mitochondrial sequences of other true bugs using Muscle as implemented in MEGA 6.06 [19]. The tRNAs were predicted by their cloverleaf secondary structure using tRNAscan-SE 1.21 [20]. Some tRNAs not detected by tRNAscan-SE were determined in the unannotated regions by sequence similarity to tRNAs of other true bugs.

Nucleotide composition and codon usage were analyzed with MEGA 6.06 [19]. The number of synonymous substitutions per synonymous site (Ks), the number of nonsynonymous substitutions per nonsynonymous site (Ka), the effective number of codons (ENC) and codon bias index (CBI) for each PCG were determined with DnaSP 5.0 [21]. Strand asymmetry was calculated using the formulas: AT-skew = [A-T]/[A+T] and GC-skew = [G-C]/[G+C] [22]. The tandem repeats of the control region were identified by tandem repeats finder online server (http://tandem.bu.edu/trf/trf.html) [23].

### Construction of rRNA Secondary Structure

The secondary structure of the large and small subunits of rRNAs (*rrnL* and *rrnS*) were inferred following the models proposed for other insects, *Drosophila melanogaster* (Diptera: Drosophilidae) [24], *Apis mellifera* (Hymenoptera: Apidae) [25], and *Manduca sexta* (Lepidoptera: Sphingidae) [26]. Helix numbering follows the convention established at the CRW site [24]. Regions lacking significant homology and other non-coding regions were folded using the Mfold Web Server [27].

### Phylogenetic Analysis

Twenty-five Pentatomomorpha species with complete or nearly complete mitogenomes were used in phylogenetic analyses, representing five superfamilies and seventeen families. Two species, *Adelphocoris fasciaticollis* and *Apolygus lucorum* from Cimicomorpha, were used as outgroups. Details of the species used in this study were listed in Table 1.

**Table 1:** List of the species included in the present study.

Infraorder	Superfamily/family	Species	Size (bp)	Accession number	Reference
Cimicomorpha	Miroidea				
	Miridae	*Adelphocoris fasciaticollis*	15,434	NC_023796	[65]
		*Apolygus lucorum*	14,768	NC_023083	[46]
Pentatomomorpha	Aradoidea				
	Aradidae	*Aradacanthia heissi*	15,528	HQ441233	[66]
		*Brachyrhynchus hsiaoi*	15,250	NC_022670	[67]
		*Neuroctenus parus*	15,354	NC_012459	[13]
	Coreoidea				
	Alydidae	*Riptortus pedestris*	17,191	NC_012462	[13]
	Coreidae	*Hydaropsis longirostris*	16,521	NC_012456	[13]
	Rhopalidae	*Corizus tetraspilus*	14,989	KM983397	This study
		*Aeschyntelus notatus*	14,532	NC_012446*	[13]
		*Stictopleurus subviridis*	15,319	NC_012888	[56]
	Lygaeoidea				
	Berytidae	*Yemmalysus parallelus*	15,747	NC_012464	[13]
	Colobathristidae	*Phaenacantha marcida*	14,540	NC_012460*	[13]
	Geocoridae	*Geocoris pallidipennis*	14,592	NC_012424*	[13]
	Lygaeidae	*Kleidocerys resedae resedae*	14,688	KJ584365	[68]
	Malcidae	*Chauliops fallax*	15,739	NC_020772	[37]
		*Malcus inconspicuus*	15,575	NC_012458	[13]
	Pentatomoidea				
	Cydnidae	*Macroscytus gibbulus*	14,620	EU427338*	[13]
	Dinidoridae	*Coridius chinensis*	14,648	JQ739179*	[69]
	Pentatomidae	*Dolycoris baccarum*	16,549	NC_020373	[47]
	Pentatomidae	*Halyomorpha halys*	16,518	NC_013272	[70]
	Pentatomidae	*Nezara viridula*	16,889	NC_011755	[13]
	Plataspidae	*Coptosoma bifaria*	16,179	NC_012449	[13]
	Plataspidae	*Megacopta cribraria*	15,647	NC_015342	Direct Submission
	Tessaratomidae	*Eusthenes cupreus*	16,229	NC_022449	[71]
	Urostylididae	*Urochela quadrinotata*	16,587	NC_020144	[72]
	Pyrrhocoroidea				
	Largidae	*Physopelta gutta*	14,935	NC_012432	[13]
	Pyrrhocoridae	*Dysdercus cingulatus*	16,249	NC_012421	[13]

*Incomplete mitochondrial genome.

Sequences of 13 PCGs (excluding stop codons), two rRNAs and 22 tRNAs were used for phylogenetic analyses. Each PCG was aligned individually with codon-based multiple alignments using MAFFT as implemented in the TranslatorX online server [28]. Gaps and ambiguous sites were removed from the protein alignment before back-translate to nucleotides using GBlocks within the TranslatorX with default settings. The rRNA genes were aligned with MAFFT (http://mafft.cbrc.jp/alignment/server/) using the Q-INS-i method [29], and poorly aligned positions and divergent regions were removed using GBlocks Server (http://molevol.cmima.csic.es/castresana/Gblocks_server.html) with allowing gap positions within the final blocks. Each tRNA was aligned using ClustalW implemented in MEGA 6.06 [19], and the resulting alignments of tRNA were carefully adjusted by eye according to the secondary structures. Alignments of individual genes were then concatenated as two datasets: 1) sequences of 13 PCGs (PCG) with 10,422 residues, and 2) sequences of 13 PCGs, 2 rRNA and 22 tRNAs (PCGRNA) with 13,406 residues. To determine if sequence saturation exists in our alignments we performed a test of substitution saturation using DAMBE 5.3.74 [30]. Saturation plots indicated that no substitution saturation was found for each data partition, even in the third position of 13 PCGs (S1 Fig). Therefore, all sites for 13 PCGs, 2 rRNAs and 22 tRNAs were used in phylogenetic analyses.

The best partitioning schemes and corresponding nucleotide substitution models for each dataset were selected by PartitionFinder 1.1.1 [31]. We created data blocks based on genes and/or codon positions, i.e. 39 partitions for the PCG dataset or 42 for the PCGRNA dataset. We used the Bayesian information criterion (BIC) and the ‘‘greedy” algorithm with branch lengths estimated as ‘‘unlinked” to search for the best-fit scheme (S2 Table). The best-fit partitioning schemes determined by PartitionFinder were implemented in the following phylogenetic analyses.

Phylogenetic analyses were performed with ML and BI methods available on the CIPRES Science Gateway 3.3 [32]. ML analysis was conducted with RAxML-HPC2 on XSEDE 8.0.24 [33] using GTRGAMMAI model, and 1000 bootstraps (BS) were used to estimate the node reliability. Bayesian analysis was carried out using MrBayes 3.2.2 [34] on XSEDE. wo independent runs with four chains (three heated and one cold) were conducted simultaneously for 10,000,000 generations, with sampling every 100 generations. Stationarity is considered to be reached when ESS (estimated sample size) value is above 100 and PSRF (potential scale reduction factor) approach 1.0 as MrBayes 3.2.2 suggested [34]. After discarding the first 25% samples as burn-in, posterior probabilities (PP) were calculated in a consensus tree.

## Results and Discussion

### Genome Organization

**Fig 1:**
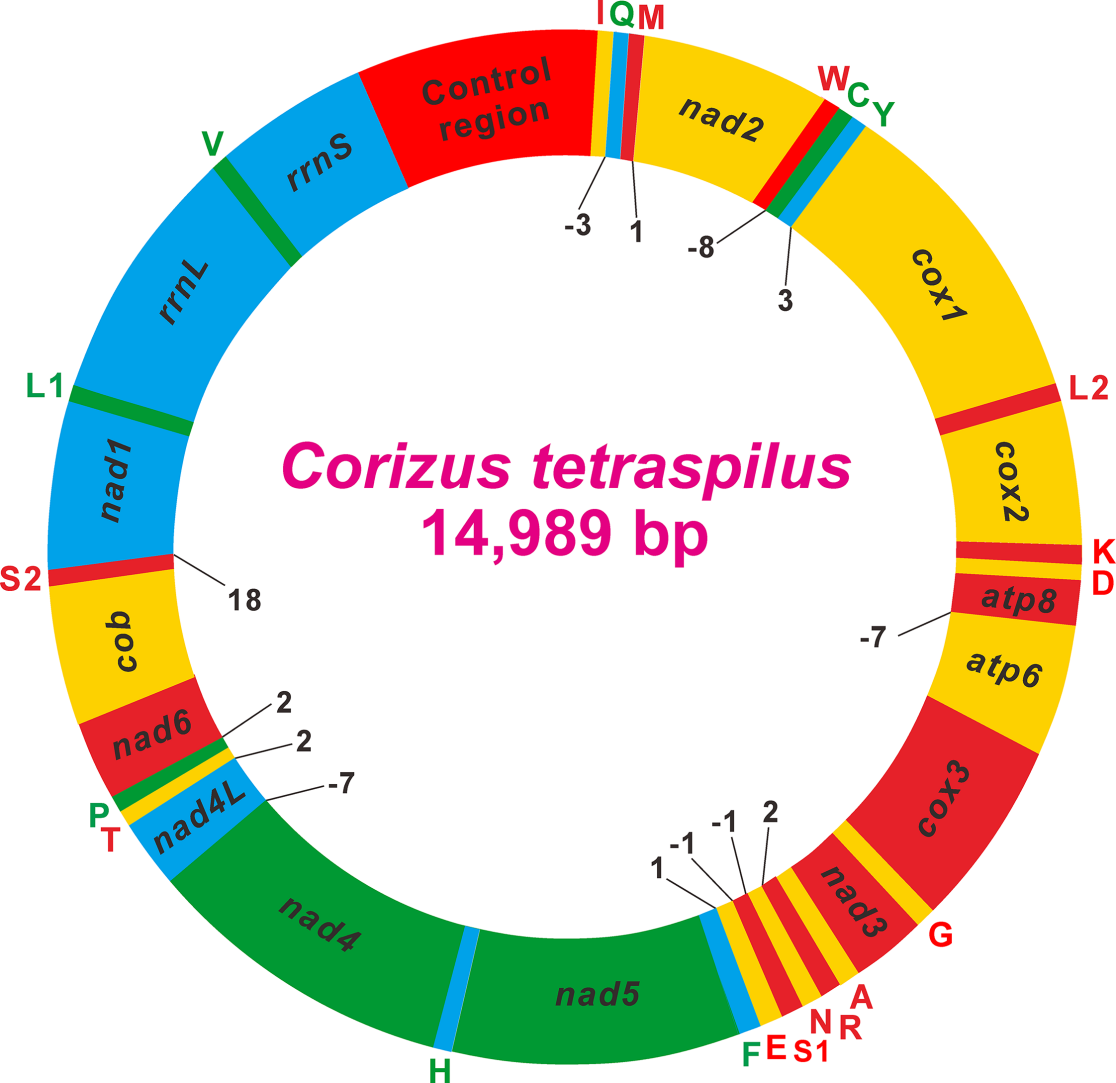
Circular map of the mitochondrial genome of *Corizus tetraspilus*. Protein coding and ribosomal genes are shown with standard abbreviations. Genes for tRNAs are abbreviated by a single letter, with S1 = AGN, S2 = UCN, L1 = CUN, and L2 = UUR. Genes coded in the J-strand (clockwise orientation) are red or orange colored. Genes coded in the N-strand (counterclockwise orientation) are green or cyan colored. Numbers at gene junctions indicate the length of small non-coding regions where negative numbers indicate overlap between genes.

The mitogenome of *C*. *tetraspilus* is a typical circular DNA molecule of 14,989 bp in size (GenBank accession no. KM983397; Fig 1, Table 2). This mitogenome is the smallest among the five sequenced Coreoidea mitogenomes (Table 1), primarily due to the significant size reduction of the putative control region. The mitogenome of *C*. *tetraspilus* contains a typical set of 37 mitochondrial genes (13 PCGs, 22 tRNA genes, 2 rRNA genes) and a large non-coding region (putative control region) (Fig 1, Table 2). The order and orientation of the mitochondrial genes is identical to that of the putative ancestral insect mitogenome [1].

**Table 2:** Annotation and organization of the complete mitogenome of *Corizus tetraspilus*.

Gene	Strand	Position	Length (bp)	Intergenic nucleotides	Start codon	Stop codon	Anticodon
*trnI*	J	1–65	65	0			GAU
*trnQ*	N	63–131	69	-3			UUG
*trnM*	J	132–200	69	0			CAU
*nad2*	J	202–1198	997	1	ATG	T	
*trnW*	J	1199–1267	69	0			UCA
*trnC*	N	1260–1322	63	-8			GCA
*trnY*	N	1323–1387	65	0			GUA
*cox1*	J	1391–2924	1534	3	TTG	T	
*trnL2*(UUR)	J	2925–2989	65	0			UAA
*cox2*	J	2990–3668	679	0	ATT	T	
*trnK*	J	3669–3742	74	0			CUU
*trnD*	J	3743–3807	65	0			GUC
*atp8*	J	3808–3963	156	0	ATA	TAA	
*atp6*	J	3957–4624	668	-7	ATG	TA	
*cox3*	J	4625–5411	787	0	ATG	T	
*trnG*	J	5412–5474	63	0			UCC
*nad3*	J	5475–5826	352	0	ATA	T	
*trnA*	J	5827–5889	63	0			UGC
*trnR*	J	5891–5950	60	1			UCG
*trnN*	J	5954–6020	67	3			GUU
*trnS1*(AGN)	J	6020–6088	69	-1			GCU
*trnE*	J	6088–6154	67	-1			UUC
*trnF*	N	6156–6222	67	1			GAA
*nad5*	N	6223–7934	1712	0	ATC	TA	
*trnH*	N	7935–7998	64	0			GUG
*nad4*	N	7999–9314	1316	0	ATG	TA	
*nad4L*	N	9308–9598	291	-7	ATT	TAA	
*trnT*	J	9601–9663	63	2			UGU
*trnP*	N	9664–9726	63	0			UGG
*nad6*	J	9729–10216	488	2	ATA	TA	
*cob*	J	10217–11348	1132	0	ATG	T	
*trnS2*(UCN)	J	11349–11417	69	0			UGA
*nad1*	N	11436–12362	927	18	ATT	TAA	
*trnL1*(CUN)	N	12363–12429	67	0			UAG
*rrnL*	N	12430–13695	1266	0			
*trnV*	N	13696–13763	68	0			UAC
*rrnS*	N	13764–14549	786	0			
control region	J	14550–14989	440	0			

The *C*. *tetraspilus* mitogenome is highly compact in genome size as that in other animals [1]. Gene overlaps have been observed at six gene junctions and involved a total of 27 nucleotides, ranging from 1 to 8 nucleotides (Fig 1, Table 1). The longest overlap (8 bp) exists between *trnW* and *trnC*, which are also present in other Coreoidea species and highly conserved with the same size (S2 Fig). Two PCG pairs *atp8*/*atp6* and *nad4L*/*nad4* overlap seven identical nucleotides in all the five Coreoidea mitogenomes (S2 Fig).

### Nucleotide Composition and Codon Usage

The nucleotide composition of the *C*. *tetraspilus* mitogenome is significantly biased toward A and T. The total A+T content of the J-strand is 74.88%, which is slightly lower than those of other completely sequenced Coreoidea species (S3 Fig). Among 13 PCGs, the lowest A+T content is 69.02% in *cox1*, while the highest is 86.27% in *atp8*. The analysis of the nucleotide composition at each codon position of the concatenated 13 PCGs of *C*. *tetraspilus* demonstrates that the third codon position (86.19%) has an A+T content higher than that of the first (70.34%) and second (67.46%) positions. The similar nucleotide composition patterns are also observed in other Coreoidea species (S3 Fig).

The *C*. *tetraspilus* mitogenome has more As and Cs, indicating a positive AT-skew (0.14) and a negative GC-skew (-0.19). The PCGs and rRNAs have a negative AT-skew and a positive GC-skew in the five Coreoidea species. For the most species of Coreoidea, values for both AT-skew and GC-skew of the second and third codon positions of PCGs are negative, whereas AT- and GC-skews of the first position are positive (S3 Fig). However, negative AT-skew of the first position and positive AT-skew of the third are found in *Hydaropsis longirostris* and *C*. *tetraspilus*, respectively, which are different from those of the other species.

**Fig 2:**
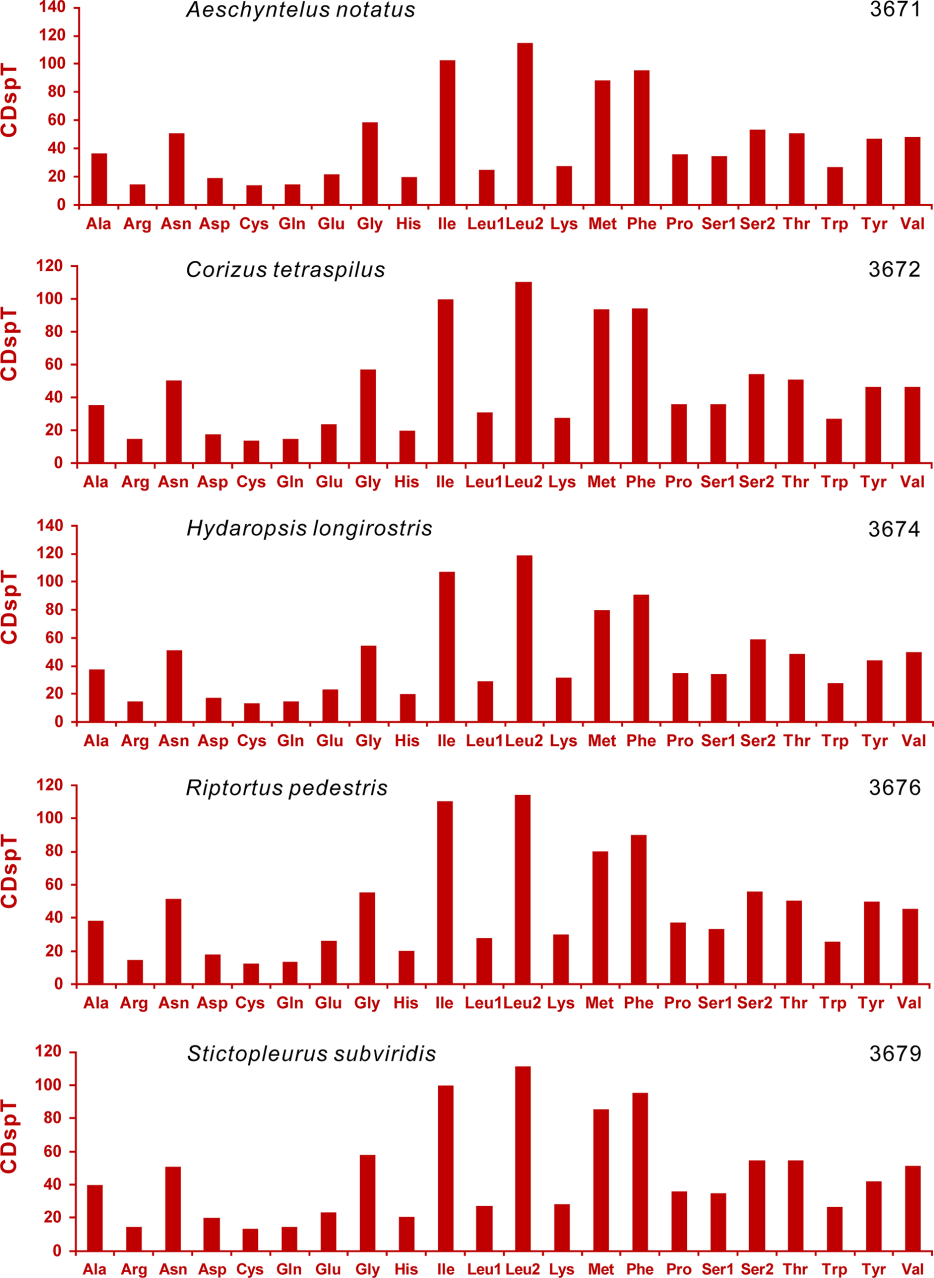
Codon distributions in the mitochondrial genomes of five Coreoidea species. Numbers to the left refer to the total number of codon. CDspT, codons per thousands codons.

**Fig 3:**
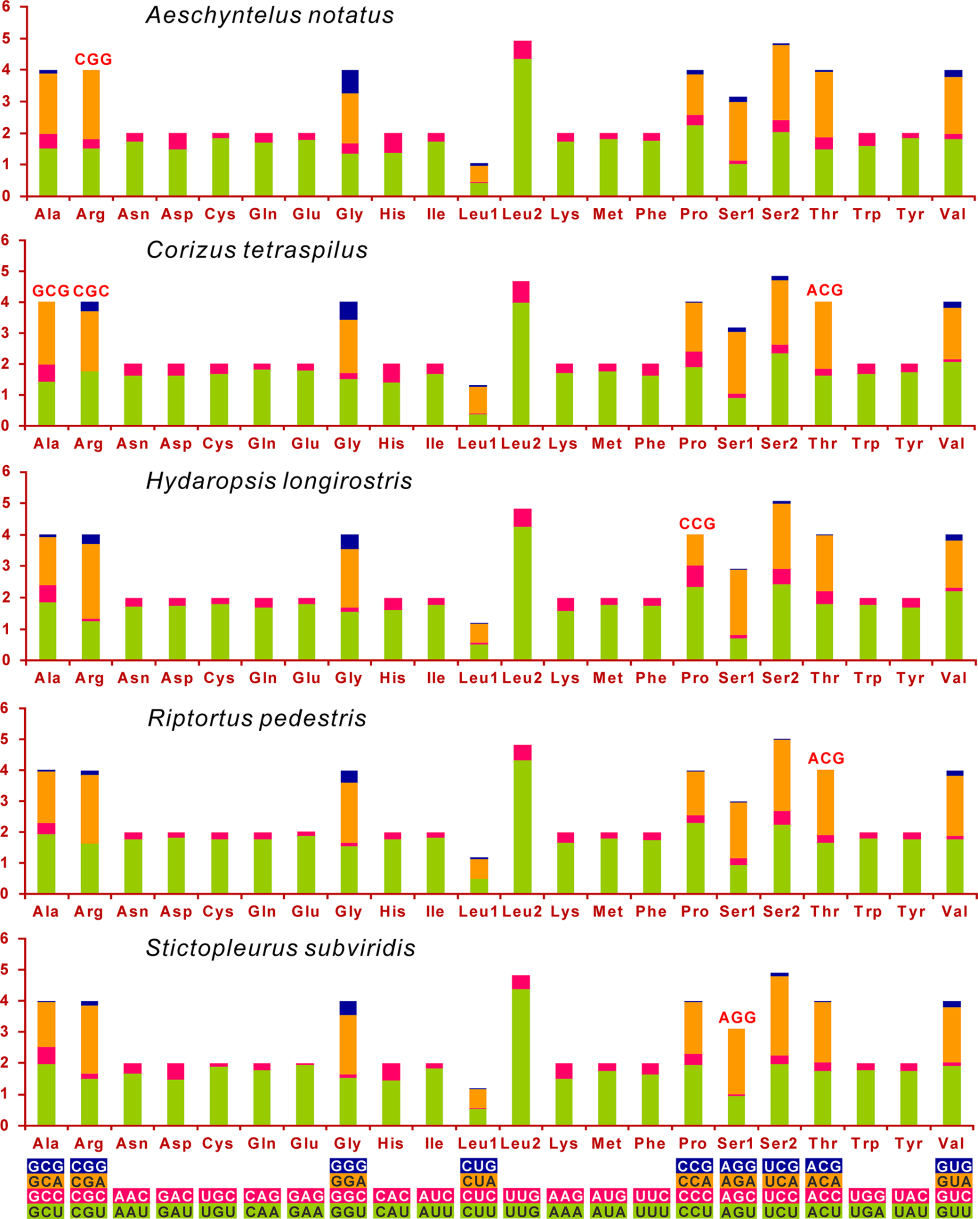
Relative Synonymous Codon Usage (RSCU) in the mitochondrial genomes of five Coreoidea species. Codons that are not present in the genome are indicated in red. Codon Families are provided on the x axis.

Excluding termination codons, the 13 PCGs in the mitogenome are composed of 3,672 codons in total (Fig 2). Approximately equivalent codon numbers are found in other four Coreoidea species, ranging from 3,671 in *Aeschyntelus notatus* to 3,679 in *Stictopleurus subviridis* (Fig 2). The codon families exhibit the same pattern of codon usage as elsewhere in the five Coreoidea species (Figs 2 and 3). The four most predominant codon families are Leu2 (UUR), Ile, Phe, and Met, each of which has at least 80 codons (CDs) per thousand CDs. For the RSCU in the mitogenomes of five Coreoidea species, the six most frequently used codons, TTT (F), TTA (L), ATT (I), ATA (M), TAT (Y) and AAT (N), are all completely composed of A and/or T, which reflects a strong compositional bias toward A+T. The four- and two-fold degenerate codon usages are biased to use more As and Ts than Gs and Cs in the third codon positions (Fig 3). Furthermore, three GC-rich codon families, i.e. GCG (A), CGC (R) and ACG (T), are not utilized in *C*. *tetraspilus*, whereas only one GC-rich codon is not used in each of other Coreoidea species (Fig 3).

The correlations between ENC, CBI, the G+C content of all codons, and the G+C content of the 3rd codon positions in all sequenced Coreoidea mitogenomes are analyzed (S4 Fig). A positive correlation is observed between ENC and G+C content of all codons (R^2^ = 0.82) (S4A Fig) and the 3rd codon positions (R^2^ = 0.94) (S4B Fig). Furthermore, a negative correlation is found between CBI and G+C content of all codons (R^2^ = 0.93) (S4C Fig), G+C content of the 3rd codon positions (R^2^ = 0.88) (S4D Fig), and ENC (R^2^ = 0.86) (S4E Fig). These results are consistent with prevailing neutral mutational theories positing that genomic G+C content is the most significant factor in determining codon bias among organisms [35, 36].

### Protein Coding Genes

Twelve of the 13 PCGs start with a typical ATN codon: one (*nad5*) with ATC, three (*cox2*, *nad4L* and *nad1*) with ATT, three (*atp8*, *nad3*, and *nad6*) with ATA, and five (*nad2*, *atp6*, *cox3*, *nad4* and *cob*) with ATG. The only exception is *cox1*, which uses TTG as a start codon. This unconventional codon has also been commonly found in the other Coreoidea mitogenomes (S3 Table) and many other true bugs [37–39]. Four PCGs (*atp6*, *atp8*, *nad1* and *nad4L*) have complete stop codon TAA, while the remaining nine terminate with either TA (*nad4*, *nad5* and *nad6*) or T (*cox1*, *cox2*, *cox3*, *cob*, *nad2* and *nad3*). Incomplete stop codons were also observed in the other Coreoidea species (S3 Table), and it has been proposed that the complete stop codon TAA could be generated via post-transcriptional polyadenylation [40, 41].

**Fig 4:**
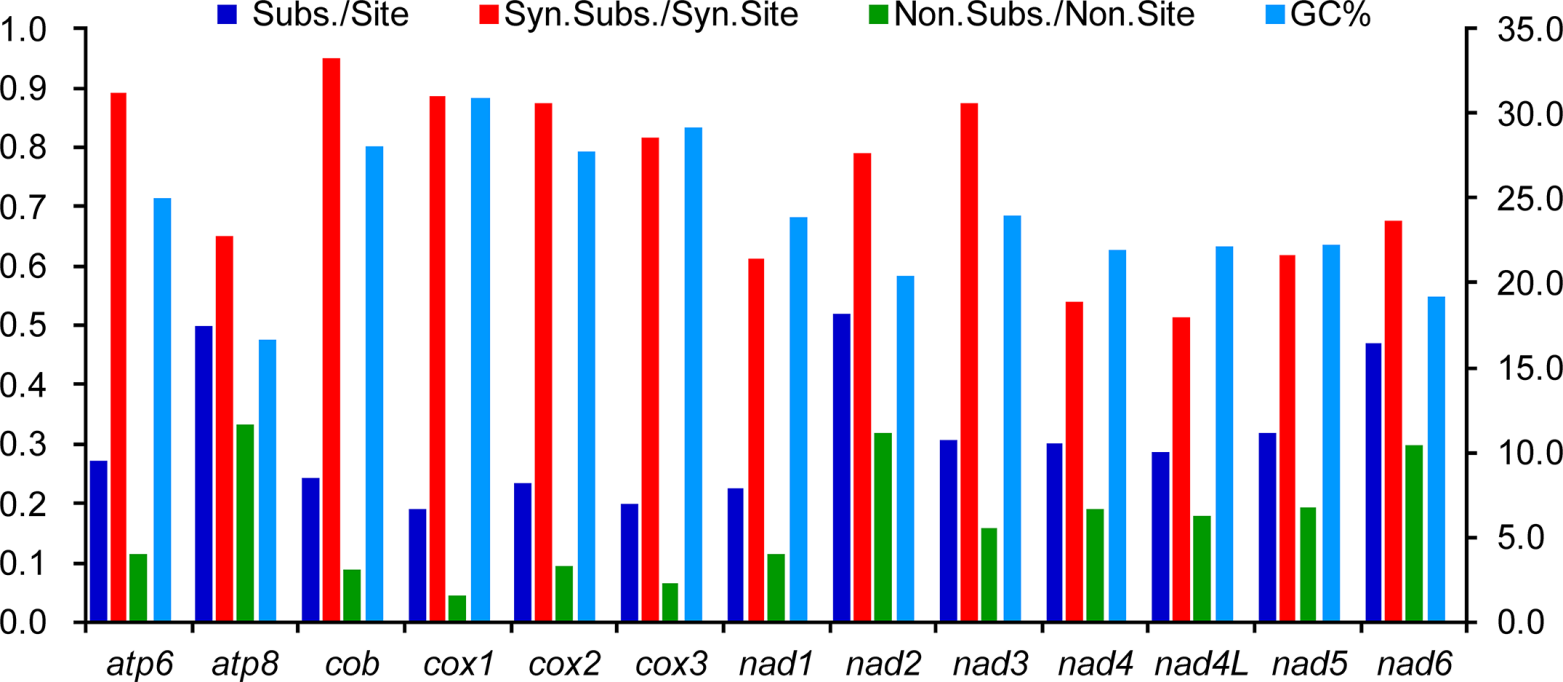
Evolutionary rates of 13 protein-coding genes in the mitochondrial genomes of five Coreoidea species.

The evolutionary patterns among the mitochondrial PCGs in Coreoidea are different (Fig 4). The Ks of *cob* is the highest, but its value of Ka is much lower, while the values of Ka and *ω* for *atp8* are the highest. The *cox1* gene has been widely used as a DNA barcode in true bugs [42–44], but this gene shows the lowest evolutionary rates, compared to other genes. Similarly, *cob*, *cox2* and *cox3* also show relatively slow revolutionary rates. By contrast, the nucleotide substation rate per site and Ka values of *nad2* and *nad6* are only lower than that of *atp8*, indicating that these two genes may be potential barcoding markers in Coreoidea. The *ω* values for all PCGs are far lower than one (< 0.52), indicating that these genes are evolving under the purifying selection. Therefore, all mitochondrial PCGs can be employed to investigate phylogenetic relationships within Coreoidea. Furthermore, a negative correlation has been found between the *ω* and the G+C content of each PCG (R^2^ = 0.93), indicating that the variation of G+C content probably causes the different evolutionary patterns among genes.

### Transfer and Ribosomal RNAs

**Fig 5:**
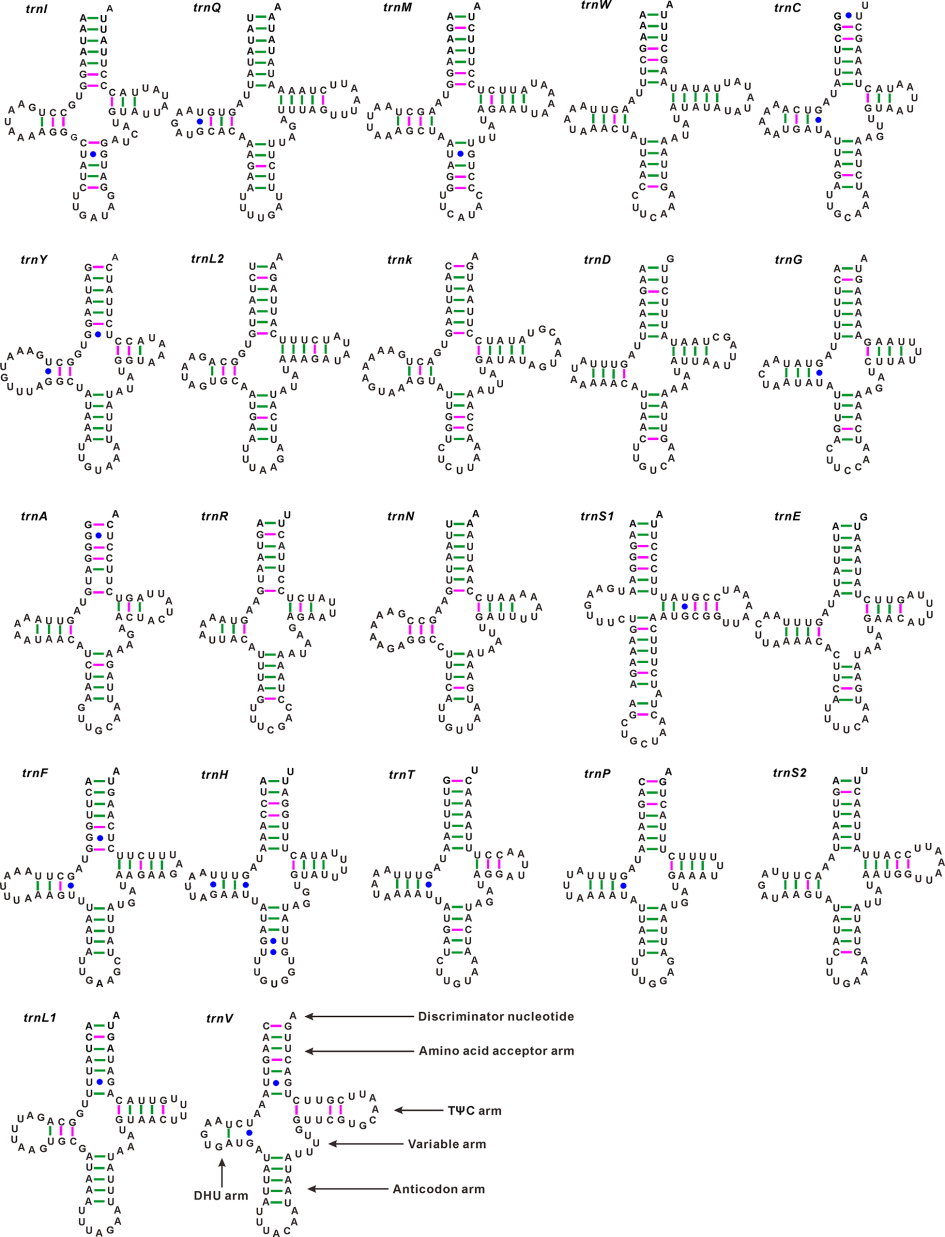
Putative secondary structures of the 22 tRNA genes identified in the mitochondrial genome of *Corizus tetraspilus*. All tRNA genes are shown in the order of occurrence in the mitochondrial genome starting from *trnI*. Bars indicate Watson–Crick base pairings, and dots between G and U pairs mark canonical base pairings in tRNA.

All the typical 22 tRNAs are found in the *C*. *tetraspilus* mitogenome, with size ranging from 60 bp to 74 bp (Table 2). The secondary structures of *C*. *tetraspilus* tRNAs are consistent with other Coreoidea species (S5 Fig). All the tRNAs could be folded into classic cloverleaf secondary structures (Fig 5), with the exception of *trnS1* (AGN) that lack the dihydrouridine (DHU) arm. The loss of the DHU arm in *trnS1* is common in insect mitogenomes [2], and has been considered a typical feature of metazoan mitogenomes. In addition, *trnS1* possesses a unusual anticodon stem (9 bp vs. the normal 5 bp) and a bulged nucleotide in the middle of the anticodon stem. Although this structure of *trnS1* is abnormal, it is highly conserved within all sequenced Coreoidea mitogenomes, especially for the anticodon arm (S5 Fig). This phenomenon found in *trnS1* has been widely reported for many other hemipterans [37, 45–48]. Furthermore, six mismatched pairs (3 U-U, 3 C-U) and 19 G-U wobble pairs are present in 7 aminoacyl acceptor stems, 10 DHU stems, 5 anticodon stems, and 3 TψC stems of the tRNA secondary structures in *C*. *tetraspilus* (Fig 5). Mismatched and wobble pairs are also detected in other Coreoidea species (S5 Fig). These mismatches are common phenomenon for invertebrate tRNAs and could be corrected by posttranscriptional RNA editing processes [49, 50].

Like other insect mitogenomes, the two genes encoding the large and small rRNA subunits (*rrnL* and *rrnS*) in *C*. *tetraspilus* are located at the conserved positions between *trnL1* (CUN) and *trnV*, and between *trnV* and the control region, respectively (Fig 1, Table 2). The ends of rRNA genes are difficult to be precisely determined by DNA sequencing alone, so they are assumed to extend to the boundaries of flanking genes [51, 52]. The *rrnL* is 1,266 bp long with an A+T content of 78.20%, and the *rrnS* is 786 bp long with an A+T content of 76.84%. The lengths and nucleotide compositions of two rRNA genes in *C*. *tetraspilus* are similar to that of other sequenced Coreoidea species (S3 Fig).

**Fig 6:**
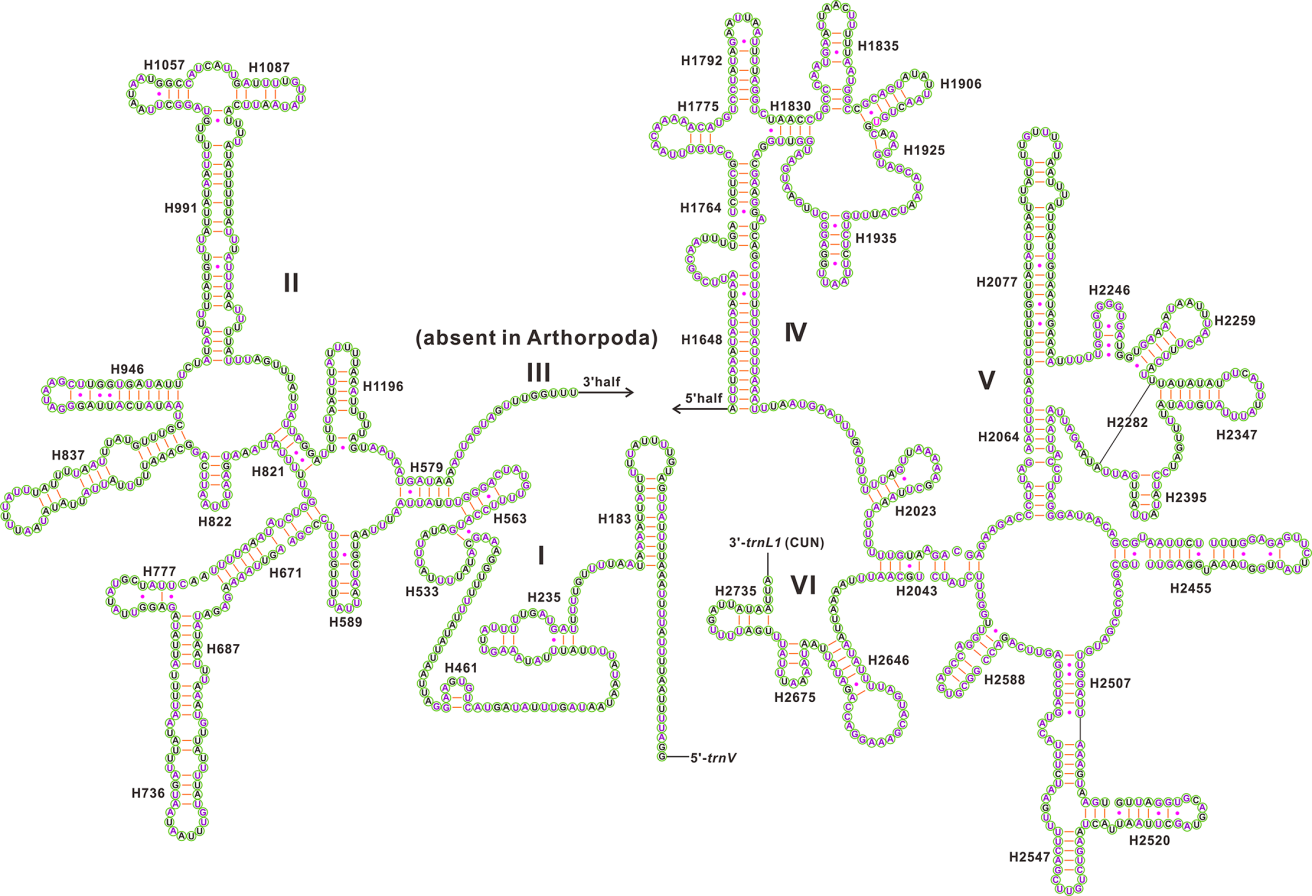
Predicted secondary structure for the *rrnL* in the mitochondrial genome of *Corizus tetraspilus*. The nucleotides showing 100% identity among sequenced Coreoidea species are marker with purple color. Inferred Watson-Crick bonds are illustrated by lines, whereas GU bonds are illustrated by dots.

**Fig 7:**
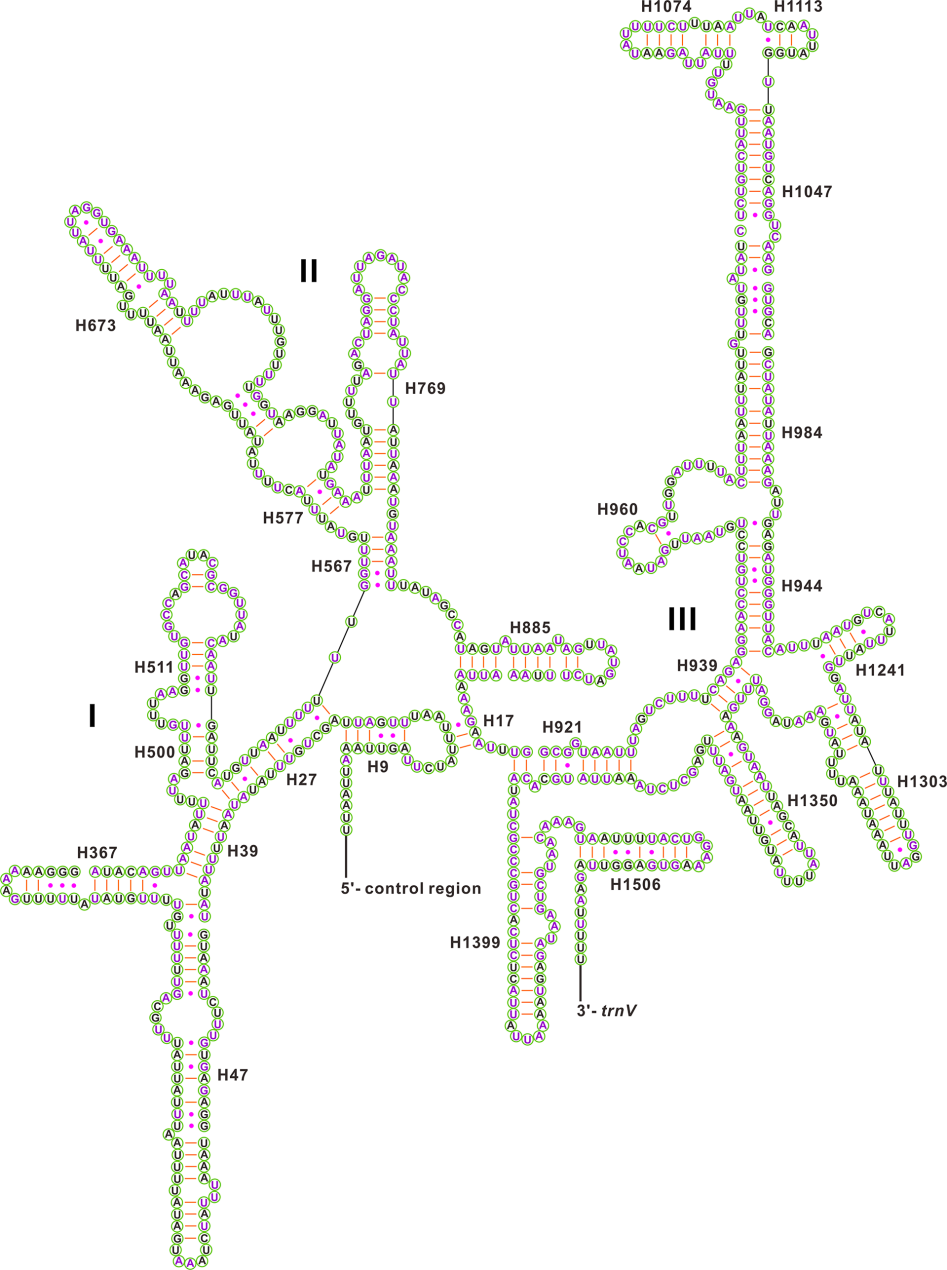
Predicted secondary structure for the *rrnS* in the mitochondrial genome of *Corizus tetraspilus*. The nucleotides showing 100% identity among sequenced Coreoidea species are marker with purple color. Inferred Watson-Crick bonds are illustrated by lines, whereas GU bonds are illustrated by dots.

The secondary structures of the two rRNA genes inferred for *C*. *tetraspilus* have similar stem-loop structures as those proposed for *Drosophila melanogaster* [24], *Apis mellifera* [25], *Manduca sexta* [26] and other hemipterans (e.g. *Chauliops fallax* [37], *Stenopirates* sp. [39] and *Cavariella salicicola* [48]). The secondary structure of *rrnL* consists of six structural domains (domain III is absent in arthropods) and 45 helices (Fig 6), whereas the secondary structure of *rrnS* contains three domains and 26 helices (Fig 7). In *rrnL*, domains IV and V are more conserved than domains I, II, and VI among sequenced Coreoidea species. Four helices (H563, H1775, H2064, H2507) of *rrnL* are most conserved with completely identical nucleotides among Coreoidea. Some helices (H183, H687, H736, H837, H991, H2077 and H2520) are greatly variable in both sequence and secondary structure among Coreoidea, as frequently observed in other insects [37, 39, 48], and their structures are inferred by the Mfold Web Server [27]. Compared to the 5’-end, the 3’-end of *rrnS* structure is more conserved among Coreoidea, especially for the helices H921-960, H1047 and H1399. The helix H47 are highly variable among different insects, and no consistent structure has been found for this region [26]. In *C*. *tetraspilus*, the possible secondary structure of this region, predicted by the Mfold Web Server [27], consists of a long stem and a short terminal loop, which is similar to that in *Stenopirates* sp. [39] and *Chauliops fallax* [37]. The helices (H1047, H1068, H1074 and H1113) are highly variable, and may yield multiple possible secondary structures due to its high A+T bias and several non-canonical base pairs as observed in other insects [25, 26, 37, 39, 48]. However, the helix H1047 is highly conserved in both sequence and structure among Coreoidea. The helix H1068 has been found in some insects [25, 26, 37, 53], but this helix seems not to be present in the *rrnS* of *C*. *tetraspilus*, which is similar to those in *Stenopirates* sp. [39] and *Cavariella salicicola* [48].

### Non-Coding Regions

The largest non-coding region (440 bp) in the *C*. *tetraspilus* mitogenome is flanked by *rrnS* and *trnI*–*trnQ*–*trnM* gene cluster (Fig 1, Table 2), and can be identified as the putative mitochondrial control region based on the conserved position compared to other insect mitogenomes. The A+T content of this region is 60.68%, which is much lower than that of the entire mitogenome, and is lowest among all the four sequenced mitochondrial control regions (S3 Fig). Although the insect mitochondrial control region is typically characterized by high A+T content, low A+T content in this region has been found in many heteropterans [13]. Furthermore, the control region of *C*. *tetraspilus* harbors more Ts than As (AT-skew = –0.12), which is opposite to that of other Coreoidea species (S3 Fig).

The length of control regions in the four completely sequenced Coreoidea mitogenomes is highly variable, ranging from 440 bp in *C*. *tetraspilus* to 1,991 bp in *H*. *longirostris*. Generally, the putative control regions of the arthropods have any or all of these four motifs: a long sequence of thymines, tandem repeats, a subregion of even higher A+T content, and stem-loop structures [54]. However, neither tandem repeats nor long T-stretches are present in Coreoidea control regions, with the exception of *Riptortus pedestris*. Although the four control regions could form several stem-loop structures, no conserved block has been found, making it difficult to identify any putatively functional motifs. No typical subregions with higher A+T content is present in the control region of *C*. *tetraspilus*, but a GC-rich region (G+C% = 76.19%) has been found at the 5’-end of the control region. A similar GC-rich region is also present in three other Coreoidea species, with G+C content ranging from 54.83% in *R*. *pedestris* to 82.60% in *S*. *subviridis*.

In addition to the putative control region, 31 nucleotides are dispersed in eight intergenic spacers, ranging in size from 1 to 18 bp (Fig 1, Table 2). The majority of intergenic spacer sequences are short (1–3 bp). The longest intergenic spacer sequence (18 bp) is located between *trnS2* (UCN) and *nad1* (Table 2). This intergenic spacer is also detected in other Coreoidea species. Similar non-coding sequences are present at this position in other insect orders [26], and these sequences have been shown to be binding site of a transcription termination factor (DmTTF) [55]. All of the sequences observed in the Coreoidea mitogenomes are highly conserved, and have a sequence of identical length (7 bp) and with significant similarity to the DmTTF binding site (S6 Fig).

In *Stictopleurus subviridis* and *R*. *pedestris*, another large non-coding region is found between *trnI* and *trnQ* [13, 56]. However, this is not true for *C*. *tetraspilus* and *H*. *longirostris*, where *trnQ* overlaps 3 nucleotides with *trnI* on the opposite strand, as found in most hemipteran mitogenomes [47, 48, 57, 58].

### Phylogenetic Relationships

**Fig 8:**
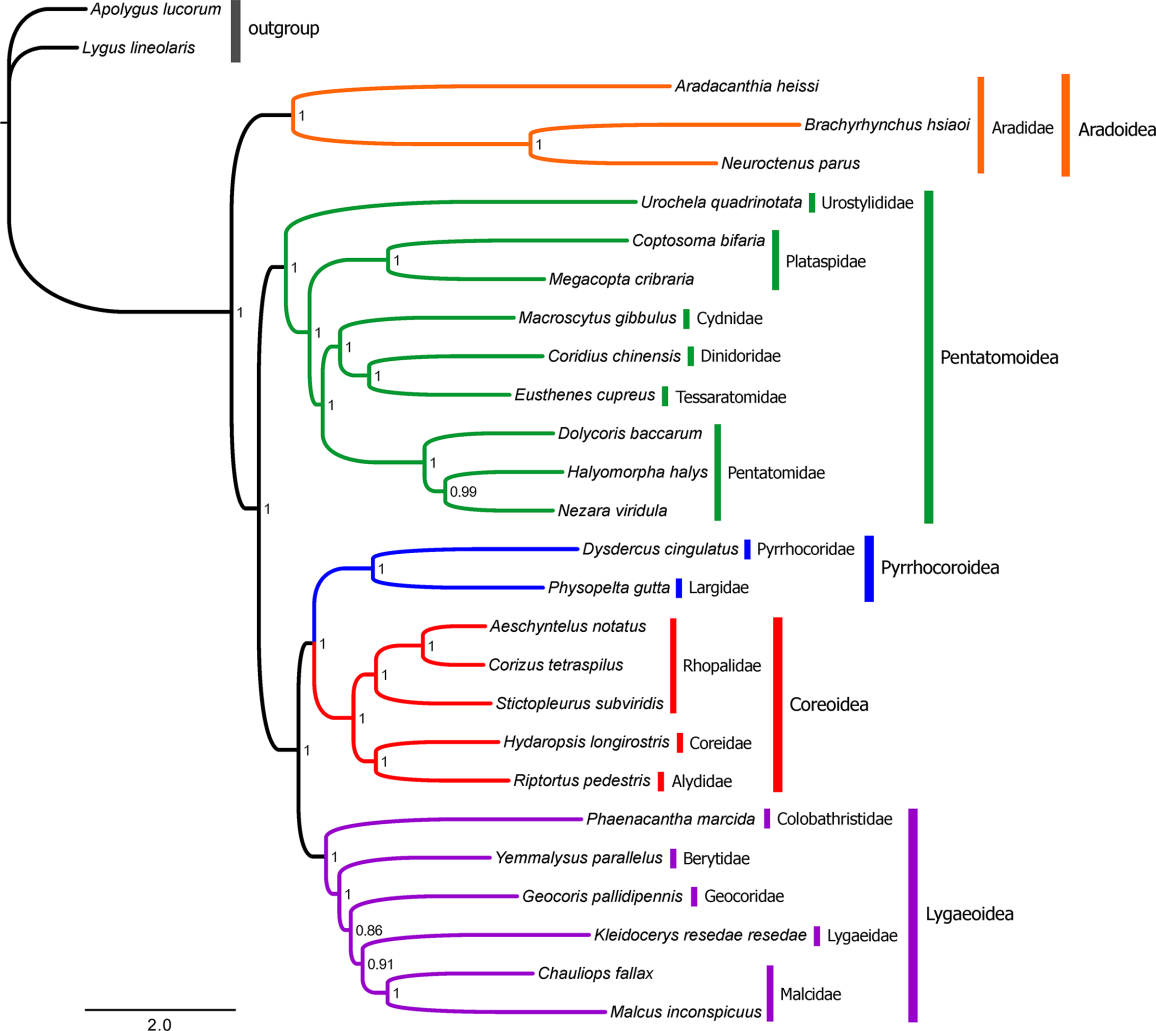
Bayesian phylogenetic relationships among five Pentatomomorpha superfamilies. Phylogenetic analysis is based on the concatenated nucleotide sequences of 13 mitochondrial protein-coding genes. Numbers on branches are Bayesian posterior probabilities.

Phylogenetic analyses based on the two datasets (PCG and PCGRNA) using two methods (BI and ML) result in almost identical tree topology (Fig 8, S7–S9 Figs). Nodal supports are generally higher in BI tree than those in ML tree generated from the same dataset, as has been revealed by previous studies [59–61]. The only topological incongruence between BI and ML trees based on sequences of 13 PCGs is the phylogenetic relationship among three species within the family Pentatomidae. In BI tree, *Nezara viridula* has a closer relationship with *Halyomorpha halys* with high supports (PP = 0.99, Fig 8), whereas a sister-species relationship between *N*. *viridula* and *Dolycoris baccarum* is recovered in ML tree with relatively low support (BS = 67, S7 Fig). Phylogenetic analyses using the PCGRNA dataset reduce support values in some nodes and the monophyly of the family Malcidae is not recovered in both BI and ML trees (S8 and S9 Figs), suggesting that RNA data might be unsuitable for reconstructing the evolutionary relationships within Pentatomomorpha.

Our results consistently recover all the superfamilies (Aradoidea, Pentatomoidea, Pyrrhocoroidea, Lygaeoidea, and Coreoidea) established previously in Pentatomomorpha as monophyletic groups with high supports (PP = 1.0, BS = 89–100; Fig 8, S7–S9 Figs). Our results also confirm the hypothesis that Aradoidea and the Trichophora are sister groups, as indicated in previous analyses based on the morphological and molecular data [13–17]. Furthermore, the sister-group relationship of Pentatomoidea and the remainder of the Trichophora is also recognized, which is congruent with previous studies [13, 14, 16, 17].

Incongruent phylogenetic relationships within Eutrichophora have been frequently observed in previous molecular studies [13, 16, 37, 62, 63]. In Eutrichophora, our study recognizes a phylogeny of (Lygaeoidea + (Pyrrhocoroidea + Coreoidea)) consistently supported by both BI and ML analyses (PP = 0.98–1.0, BS = 52–100; Fig 8, S7–S9 Figs), which is consistent with traditional taxonomic hypotheses based on morphology [17] and molecular phylogenetic studies [16]. Especially, this relationship is also recognized by previous studies base on mitogenome data [37, 62]. However, our results are different from that of [13] where the sister-relationship between Lygaeoidea and Coreoidea was revealed based on mitogenomic data. This conflict relationship within Eutrichophora may be due to different taxa sampling and analytical methods. A total of 13 taxa from Pentatomomorpha were used in [13], while 25 species from Pentatomomorpha are included in the present study. The number of species included in Eutrichophora has increased from seven species analyzed by [13] to 13 species used in our study. In [13] all alignments were performed with Clustal W, whereas in the present study rRNA genes are aligned with MAFFT (Q-INS-i method) which has been shown to be more accurate than other programs due to considering the secondary structures of rRNA [29]. For resulting alignment of each gene, poorly aligned positions and divergent regions are removed using GBlocks in our study, but not removed in [13]. In addition, we use PartitionFinder to find both the best partitioning strategy and models of substitution for each partition in Bayesian and ML analyses, whereas in [13] phylogenetic analyses were conducted with a GTR+I+G model without data partitions. The partitioning strategy might optimize the information from the genes and codon positions, which markedly improves phylogenetic resolution in recent studies [60, 64]. Although the present study based on the limited taxa is difficult to well infer the family level relationships within each superfamilies, it still has important implications for the usefulness of mitogenome sequences in evolutionary and phylogenetic studies of Pentatomomorpha.

## Supporting Information

S1 Fig Substitution saturation of 13 protein-coding genes (PCGs), 2 rRNA genes (*rrnL* and *rrnS*) and 22 RNA genes. Transitions and transversions plotted against the F84 distance. (A) first codon positions of 13 PCGs; (B) second codon positions of 13 PCGs; (C) third codon positions of 13 PCGs; (D) all sites of 13 PCGs; (E) all sites of *rrnL*; (F) all sites of *rrnS*; and (G) all sites of tRNAs.
(PDF)

S2 Fig Alignment of the three longest gene overlaps among the mitochondrial genomes of five Coreoidea species. (TIF)

S3 Fig Nucleotide composition of mitochondrial genomes of five Coreoidea species. (TIF)

S4 Fig Evaluation of codon bias in the mitochondrial genomes of five Coreoidea species. Species are abbreviated as following: AN, *Aeschyntelus notatus*; CT, *Corizus tetraspilus*; HL, *Hydaropsis longirostris*; RP, *Riptortus pedestris*; SS, *Stictopleurus subviridis*.
(TIF)

S5 Fig Alignment of the 22 mitochondrial tRNA genes in five Coreoidea species. See S4 Fig for the full names of species.
(TIF)

S6 Fig Sequence alignments of non-coding region (between *trnS2* and *nad1*) between five Coreoidea species and *Drosophila melanogaster*. (TIF)

S7 Fig Maximum likelihood tree among five Pentatomomorpha superfamilies inferred from the concatenated nucleotide sequences of 13 mitochondrial protein-coding genes. Numbers on branches are bootstrap support values.
(PDF)

S8 Fig Bayesian phylogenetic tree among five Pentatomomorpha superfamilies inferred from the concatenated nucleotide sequences of 13 mitochondrial protein-coding genes and 24 RNA genes. Numbers on branches are Bayesian posterior probabilities.
(PDF)

S9 Fig Maximum likelihood tree among five Pentatomomorpha superfamilies inferred from the concatenated nucleotide sequences of 13 mitochondrial protein-coding genes and 24 RNA genes. Numbers on branches are bootstrap support values.
(PDF)

S1 Table Primers used in this study. (DOCX)

S2 Table The best partitioning scheme selected by PartitionFinder for the concatenated nucleotide sequences of 13 protein-coding genes. (DOCX)

S3 Table Start and stop codons of mitochondrial protein-coding genes of Coreoidea. (XLSX)
