# Supplementary material for: The Complete Mitochondrial Genome of Corizus tetraspilus (Hemiptera: Rhopalidae) and Phylogenetic Analysis of Pentatomomorpha
Source: PLoS One. 2015 Jun 4;10(6):e0129003. doi: 10.1371/journal.pone.0129003 (PMC4456165; doi:10.1371/journal.pone.0129003)
Supplement: S1 Fig — Transitions and transversions plotted against the F84 distance. (A) first codon positions of 13 PCGs; (B) second codon positions of 13 PCGs; (C) third codon positions of 13 PCGs; (D) all sites of 13 PCGs; (E) all sites of rrnL; (F) all sites of rrnS; and (G) all sites of tRNAs. (PDF) [file pone.0129003.s001.pdf]

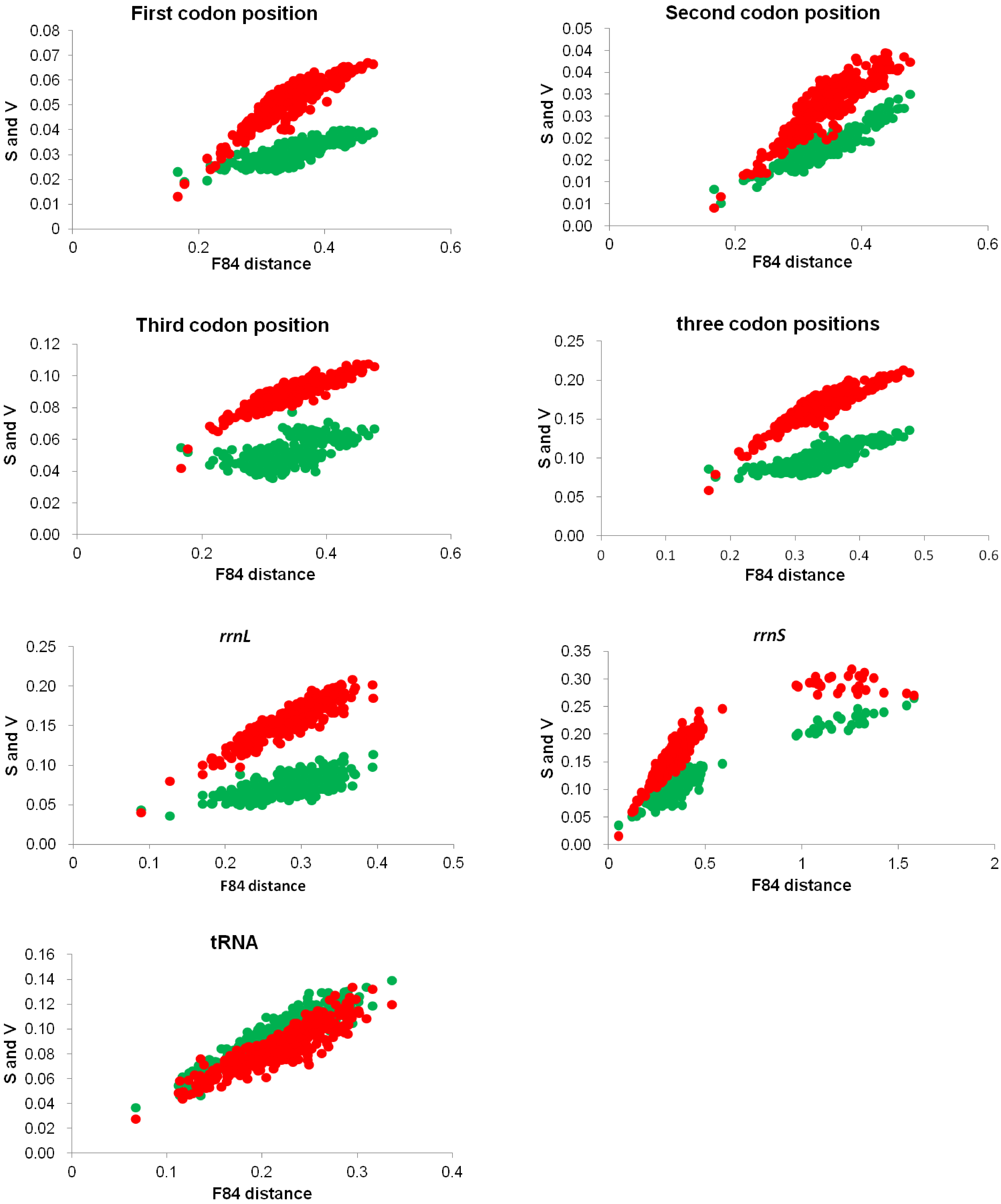
First codon position
S and V
F84 distance
0.08
0.07
0.06
0.05
0.04
0.03
0.02
0.01
0
0
0.2
0.4
0.6
Second codon position
S and V
F84 distance
0.05
0.04
0.04
0.03
0.03
0.02
0.02
0.01
0.01
0.00
0
0.2
0.4
0.6
Third codon position
S and V
F84 distance
0.12
0.10
0.08
0.06
0.04
0.02
0.00
0
0.2
0.4
0.6
three codon positions
S and V
F84 distance
0.25
0.20
0.15
0.10
0.05
0.00
0
0.1
0.2
0.3
0.4
0.5
0.6
rrnL
S and V
F84 distance
0.25
0.20
0.15
0.10
0.05
0.00
0
0.1
0.2
0.3
0.4
0.5
rrnS
S and V
F84 distance
0.35
0.30
0.25
0.20
0.15
0.10
0.05
0.00
0
0.5
1
1.5
2
tRNA
S and V
F84 distance
0.16
0.14
0.12
0.10
0.08
0.06
0.04
0.02
0.00
0
0.1
0.2
0.3
0.4
