# Supplementary figures and images for: The Complete Mitochondrial Genome of Corizus tetraspilus (Hemiptera: Rhopalidae) and Phylogenetic Analysis of Pentatomomorpha
Source: PLoS One. 2015 Jun 4;10(6):e0129003. doi: 10.1371/journal.pone.0129003 (PMC4456165; doi:10.1371/journal.pone.0129003)

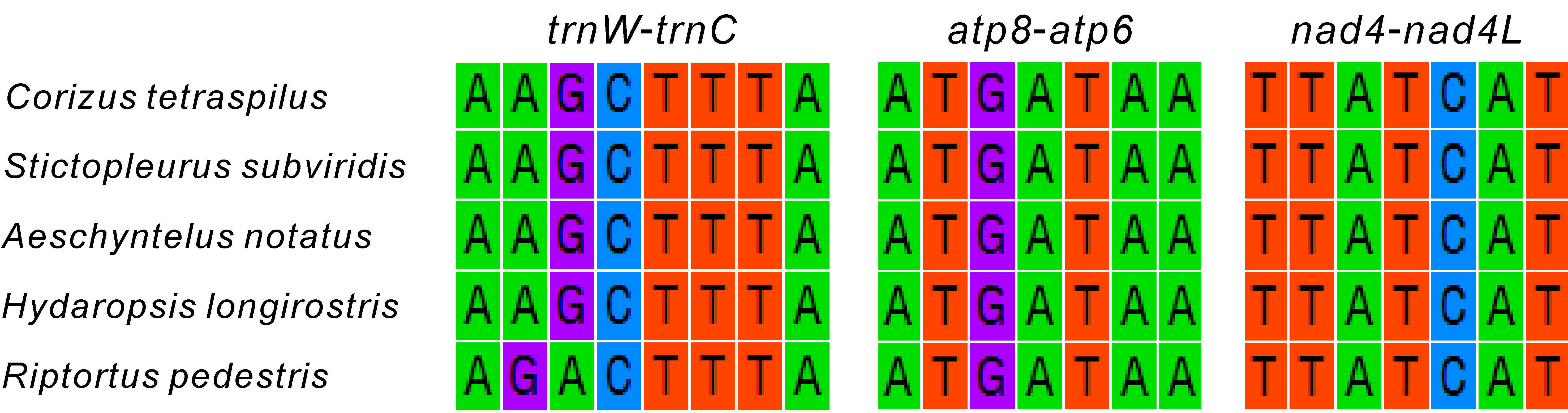

Supplement: S2 Fig — (TIF) [file pone.0129003.s002.tif]

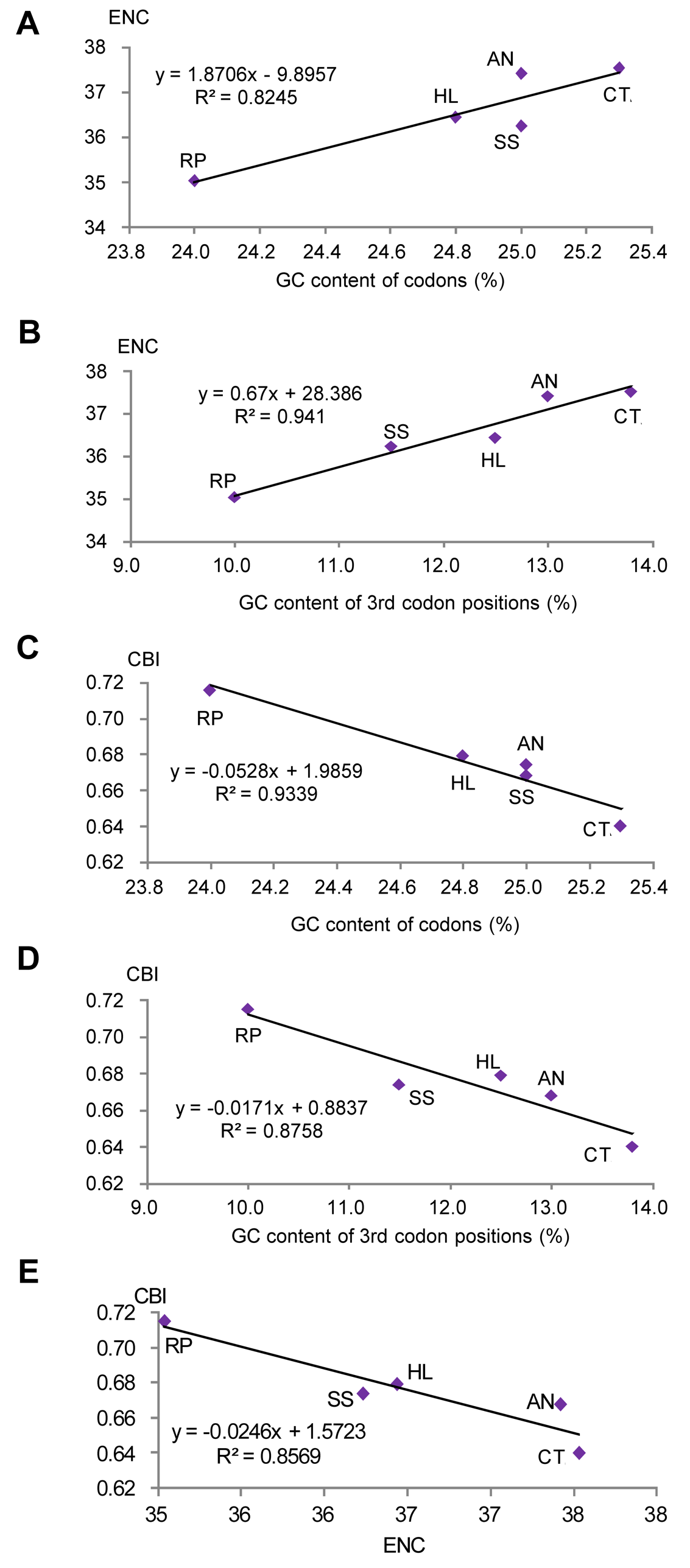

Supplement: S4 Fig — Species are abbreviated as following: AN, Aeschyntelus notatus; CT, Corizus tetraspilus; HL, Hydaropsis longirostris; RP, Riptortus pedestris; SS, Stictopleurus subviridis. (TIF) [file pone.0129003.s004.tif]

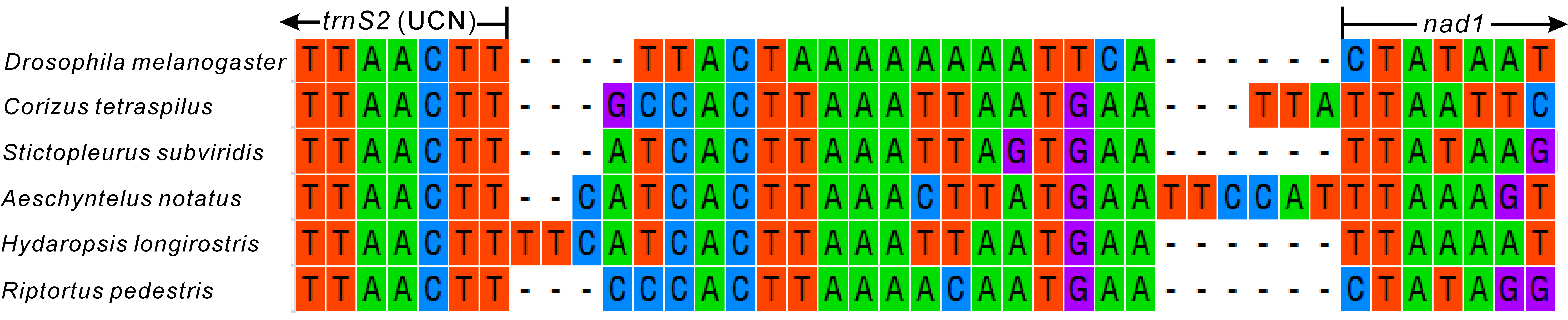

Supplement: S6 Fig — (TIF) [file pone.0129003.s006.tif]

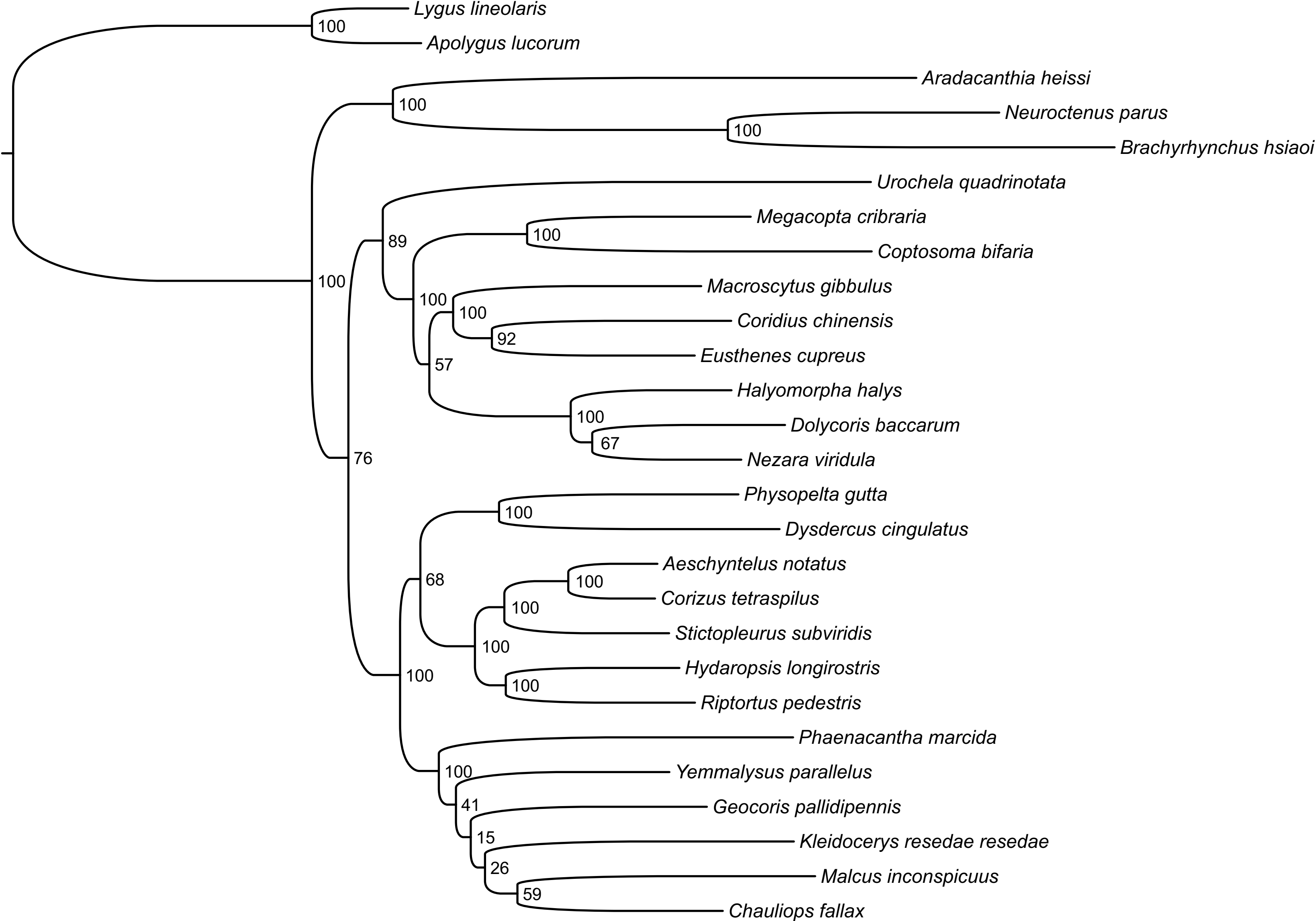

2.0

Supplement: S7 Fig — Numbers on branches are bootstrap support values. (PDF) [file pone.0129003.s007.pdf]

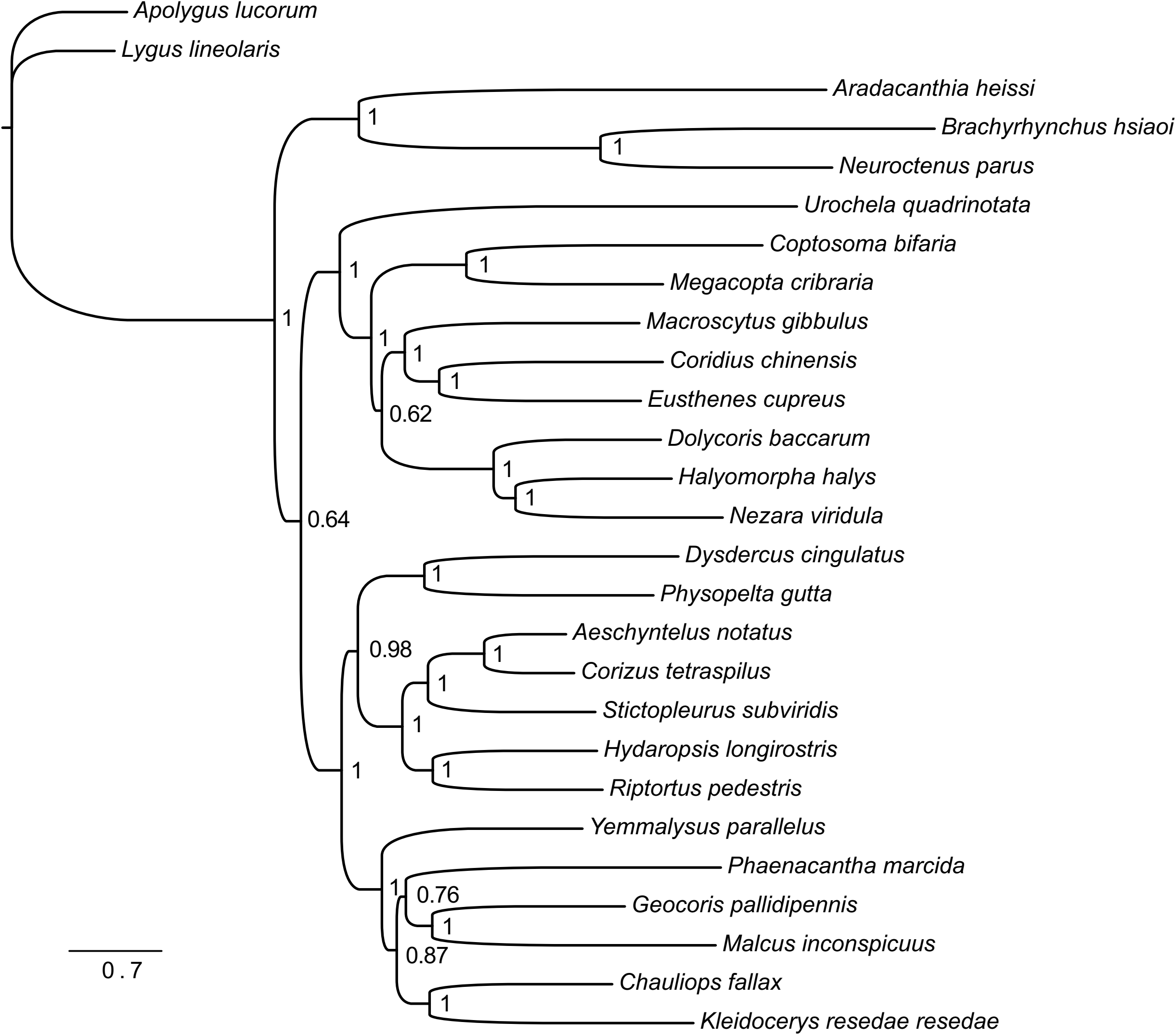

Supplement: S8 Fig — Numbers on branches are Bayesian posterior probabilities. (PDF) [file pone.0129003.s008.pdf]

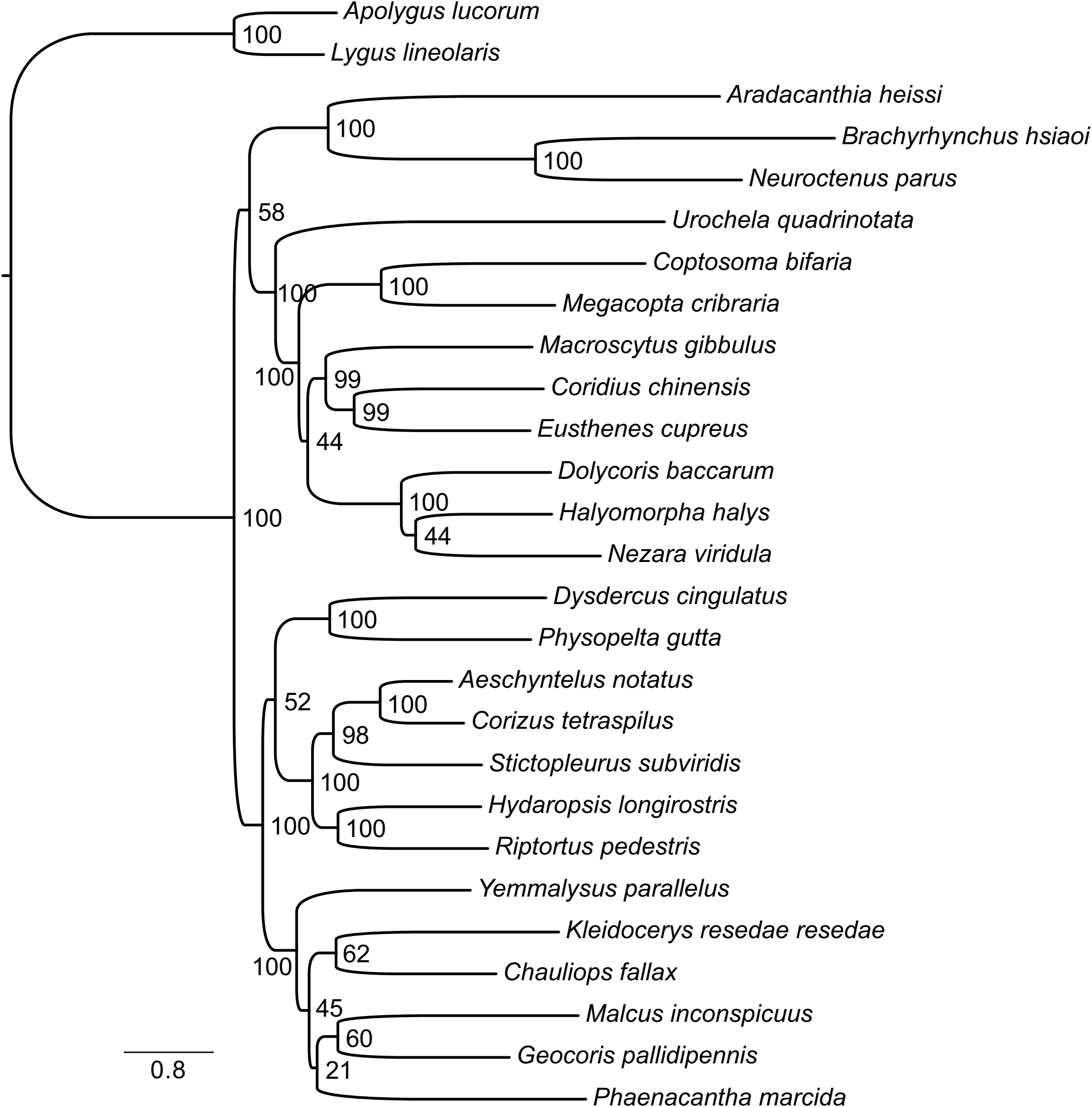

Supplement: S9 Fig — Numbers on branches are bootstrap support values. (PDF) [file pone.0129003.s009.pdf]
