# Supplementary material for: The Complete Mitochondrial Genome of Corizus tetraspilus (Hemiptera: Rhopalidae) and Phylogenetic Analysis of Pentatomomorpha
Source: PLoS One. 2015 Jun 4;10(6):e0129003. doi: 10.1371/journal.pone.0129003 (PMC4456165; doi:10.1371/journal.pone.0129003)
Supplement: S1 Table — (DOCX) [file pone.0129003.s010.docx]

**Table S1** Primer sequences used to amplify the mitogenome of *Corizus* *albomarginatus.*

| No. fragment | Name | Sequences (5'-3') | Reference |
| --- | --- | --- | --- |
| 1 | TM-J210 | AATTAAGCTATTAGGTTCATACCC | Simon et al., 2006 |
|  | TW-N1284 | TTAACTTTGAAGGTTAATAGTTT |  |
| 2 | LEM-F1 | CCTCCATTTCTAGGATTTTTACCC | this study |
|  | LED-R1 | CTGTTCATCCAGTCCCTGCT |  |
| 3 | C1-J1709 | AATTGGWGGWTTYGGAAAYTG | Simon et al., 2006 |
|  | C1-N2776 | GATAATCTGAGTATCGWCGNGG |  |
| 4 | LED-F1 | TCACAATTGGGGGTTTAACA | this study |
|  | LEN-R1 | TGGCCTTGGTCTTGTCTCTT |  |
| 5 | N5-J7572 | AAAGGGAATTTGAGCTCTTTTWGT | Simon et al., 2006 |
|  | N4-N8727 | AAATCTTTRATTGCTTATTCWTC |  |
| 6 | LEN-F1 | AGTTCTCCACCCCACAATTC | this study |
|  | LEH-R1 | AACGTGTCAGAGTTGCGTTG |  |
| 7 | CB-J10933 | GTTCTACCTTGAGGNCAAATRTC | Simon et al., 2006 |
|  | N1-N12595 | GTWGCTTTTTTAACTTTATTRGARCG |  |
| 8 | N1-J12261 | TACTTCATAAGAAATAGTYTGRGC | Simon et al., 2006 |
|  | LR-N13000 | TTACCTTAGGGATAACAGCGTAA |  |
| 9 | LR-J12888 | CCGGTTTGAACTCARATCATGTA | Simon et al., 2006 |
|  | SR-N14220 | ATATGYACAYATTGCCCGTC |  |
| 10 | LEJ-F1 | TCAGAACGAATTGCACGAAC | this study |
|  | LEM-R1 | TCCATATGCCTATCCAGTTGT |  |
