# Supplementary material for: The Complete Mitochondrial Genome of Corizus tetraspilus (Hemiptera: Rhopalidae) and Phylogenetic Analysis of Pentatomomorpha
Source: PLoS One. 2015 Jun 4;10(6):e0129003. doi: 10.1371/journal.pone.0129003 (PMC4456165; doi:10.1371/journal.pone.0129003)
Supplement: S2 Table — (DOCX) [file pone.0129003.s011.docx]

**Table S2** The best partitioning scheme selected by PartitionFinder for the two datasets used in the phylogenetic analyses.

| **Dataset** | **Subset** | **Subset Partitions** | **Best Model** |
| --- | --- | --- | --- |
| PCG | P1 | atp6_pos1, atp8_pos1, nad2_pos1, nad3_pos1, nad6_pos1 | GTR+I+G |
|  | P2 | atp6_pos2, atp8_pos2, cob_pos2, cox1_pos2, cox2_pos2, cox3_pos2, nad1_pos2, nad2_pos2, nad3_pos2, nad4L_pos2, nad4_pos2, nad5_pos2, nad6_pos2 | GTR+I+G |
|  | P3 | atp6_pos3, atp8_pos3, cob_pos3, cox1_pos3, cox2_pos3, cox3_pos3, nad2_pos3, nad3_pos3, nad6_pos3 | GTR+I+G |
|  | P4 | cob_pos1, cox1_pos1, cox2_pos1, cox3_pos1 | GTR+I+G |
|  | P5 | nad1_pos1, nad4L_pos1, nad4_pos1, nad5_pos | GTR+I+G |
|  | P6 | nad1_pos3, nad4L_pos3, nad4_pos3, nad5_pos3 | GTR+I+G |
| PCGRNA | P1 | atp6_pos1, atp8_pos1, nad2_pos1, nad3_pos1, nad6_pos1 | GTR+I+G |
|  | P2 | atp6_pos2, atp8_pos2, cob_pos2, cox1_pos2, cox2_pos2, cox3_pos2, nad1_pos2, nad2_pos2, nad3_pos2, nad4L_pos2, nad4_pos2, nad5_pos2, nad6_pos2 | GTR+I+G |
|  | P3 | atp6_pos3, atp8_pos3, cob_pos3, cox1_pos3, cox2_pos3, cox3_pos3, nad2_pos3, nad3_pos3, nad6_pos3 | GTR+I+G |
|  | P4 | cob_pos1, cox1_pos1, cox2_pos1, cox3_pos1, tRNA | GTR+I+G |
|  | P5 | nad1_pos1, nad4L_pos1, nad4_pos1, nad5_pos1 | GTR+I+G |
|  | P6 | nad1_pos3, nad4L_pos3, nad4_pos3, nad5_pos3 | GTR+I+G |
|  | P7 | rrnL | GTR+I+G |
|  | P8 | rrnS | GTR+G |
